# Adoption of practice guidelines and assessment tools in substance abuse treatment

**DOI:** 10.1186/1747-597X-5-4

**Published:** 2010-03-26

**Authors:** Traci Rieckmann, Bret E Fuller, Goal Auzeen Saedi, Dennis McCarty

**Affiliations:** 1 Department of Public Health and Preventive Medicine, Oregon Health & Science University, 3181 SW Sam Jackson Park Rd, Mailcode CB669, Portland, OR 97239 USA; 2 Portland VA Medical Center, 3710 SW U.S. Veterans Hospital Road, Mailcode P3MHDC, Portland, OR 97239 USA; 3 Department of Psychology, University of Notre Dame, 118 Haggar Hall, Notre Dame, IN 46556 USA

## Abstract

**Background:**

The gap between research and practice limits utilization of relevant, progressive and empirically validated strategies in substance abuse treatment.

**Methods:**

Participants included substance abuse treatment programs from the Northeastern United States. Structural equation models were constructed with agency level data to explore two outcome variables: adoption of practice guidelines and assessment tools at two points in time; models also included organizational, staffing and service variables.

**Results:**

In 1997, managed care involvement and provision of primary care services had the strongest association with increased use of assessment tools, which, along with provision of counseling services, were associated with a greater use of practice guidelines. In 2001, managed care involvement, counseling services and being a stand-alone drug treatment agency were associated with a greater use of assessment tools, which was in turn related to an increase in the use of practice guidelines.

**Conclusions:**

This study provides managers, clinicians and policy-makers with a framework for understanding factors related to the adoption of new technologies in substance abuse treatment.

## Background

A "widespread unease with the slow pace of adoption of research findings" has developed in the United States field of alcohol and drug treatment [1]. Simultaneously, the complexity of treatment needs, and pressure for high quality services for fewer treatment dollars, has increased the need for educated and highly skilled clinicians. However, the "hodgepodge" of funding streams and lack of formal training in the treatment of alcohol and drug disorders at the graduate level prevent some counselors and agencies from adopting innovations [2]. One such area in which this gap exists is the adoption of practice guidelines that incorporate the use of standardized assessment tools and empirically supported treatments for substance abuse treatment.

### Science-based Practice Tools and Guidelines

Variations in practice styles, inappropriate and unnecessary use of services, uncertain health outcomes and risk management have all been linked to the increased need for outcome and effectiveness research and application of science-based practice strategies and guidelines [3]. In contrast, others assert that practice guidelines and manualized treatment tools may "constrain clinical decision-making" [4], and often are based on questionable methods and limited findings. According to Weisz and colleages, the proliferation of collectively produced guidelines represents an effort to increase order and coherence through convention, standards, and regulation of the rapidly expanding and heterogenous medical domain [5]. Thus, rising health care costs and standardization of performance and outcome measurement have increased the development of assessment tools, manualized treatments, and evidence-based strategies available for substance abuse treatment. Practice guidelines that incorporate empirically supported treatments are described as systematically developed statements which assist decision-making and are suggestive, rather than prescriptive, in terms of identifying and providing efficacious treatment recommendations [3,4,6,7].

Currently, there are several practice guidelines for substance abuse treatment. The American Psychiatric Association suggests six components of psychiatric management: (1) the establishment and maintenance of a therapeutic relationship, (2) continued monitoring of the patient's clinical status, (3) management of states of intoxication and withdrawal, (4) reduction of morbidity, (5) facilitation of treatment plan adherence and provision of educational materials, and finally (6) diagnosis and treatment of psychiatric disorders [8]. Another important practice guideline or decision-making algorithm is the American Society of Addiction Medicine (ASAM) level of care criteria. ASAM published this first set of placement criteria in 1991 [9], and they have revised and improved upon the model with the second edition, the Patient Placement Criteria (PPC-2R), published in April 2001 [10]. As one of the most widely used guidelines for the placement and continued stay and subsequent discharge of substance abuse patients, the ASAM criteria is a common acronym across settings in substance abuse service delivery. In regular use within a number of treatment settings, the ASAM PPC-2R provides criteria for five levels of care for adults and adolescents: Level 0.5, Early Intervention; Level I, Outpatient Treatment; Level II, Intensive Outpatient/Partial Hospitalization; Level III, Residential/Inpatient Treatment; and Level IV, Medically-Managed Intensive Inpatient Treatment. Data collected from all 51 single state authorities (SSAs) for substance abuse treatment suggest that ASAM is in widespread use (T.R., unpublished data). SSA representatives from each of the 50 states and Washington DC were interviewed in 2009. SSA representatives used a Likert-type scale ("1 = not at all" to "5 = extensively") to rate the extent to which their state had implemented ASAM. Nearly all SSAs (86.2%) have implemented ASAM at least "slightly", with mean nationwide implementation of 4.4 (out of 5) in 2009.

Another comprehensive effort in establishing practice guidelines for substance abuse treatment has been made by the United States Substance Abuse and Mental Health Services Administration's Center for Substance Abuse Treatment's (CSAT) Treatment Improvement Protocol (TIP) series. Guidelines for treatment are based on an extensive review of the literature and are available in easy to access manuals for specific populations, diagnostic cohorts or co-occurring disorders. Finally, the American Psychological Association took an even broader view related to practice guidelines and developed criteria for the development and evaluations of such guidelines to support clinicians and organizations as they make decisions about implementation of practice guidelines [11]. Thus, there are several key decision-making guidelines and resources across the service delivery system.

Research supports the real-world efficacy of following research-derived practice guidelines in substance abuse treatment. Two examples are opiate substitution treatment for heroin dependence [12] and buprenorphine for acute heroin detoxification [13]. Providing managers, clinicians and policy-makers with a framework for understanding organizational, staffing and service delivery factors related to the adoption of new technologies is critical in an era of increased accountability. To better conceptualize these constructs several models exploring diffusion have been developed and preliminary studies are emerging in the substance abuse treatment literature.

### Models of Adoption and Diffusion

Simpson's conceptual framework suggests that institutional/personal readiness and organizational dynamics predict exposure, adoption, implementation, and routine use of innovative practices including practice guidelines [14]. The model delineates interrelationships among readiness for change, training, staff attributes, and institutional resources and support. Successful technology transfer efforts make use of interpersonal contacts (consultants, researchers on location, multi-site workshops) [1]. Thomas and colleagues [15] offer a framework based on classical diffusion theory [16] and posit that environmental factors and clinicians affect the adoption of new technologies or strategies. This model includes interactions between clinician characteristics (age, training, experience), organizational or practice characteristics (philosophy, mission, financing, structure), qualities of the new technology, the patient population, and the treatment environment (state policies, market environment, disease prevalence). These models posit that organizational characteristics and staffing patterns may influence the adoption of new innovations.

Practitioners with more formal education, and with higher social status, tend to have a more favorable outlook regarding change [17]. Adopters also are more likely to seek out information and have national professional contacts [17]. Organizational structure also can affect adoption of new practice and guidelines. Structural characteristics may include the organization's location, size, accreditation, and overall mission [18]. The mix of funding streams, and the related requirements for each entity that programs derive reimbursement or revenue from, is also a major structural or organizational factor influencing use of specific practices. Agencies that have managed care contracts are likely to be encouraged to standardize their practices and use of tools as the managed care companies seek to match requirements and reports across their networks. Organizational dynamics, climate, and the readiness to change are also integral components of the adoption of new technologies or practices [14]. Finally, Fixsen and colleagues developed a cycle of core components ("implementation drivers") including staff selection, training, consultation, and coaching (*e.g*., providing supervision, feedback, and emotional support) to frame the dynamic process of successful implementation [19].

Staff factors also may determine whether a new assessment tool or practice guideline is adopted into clinical practice. An examination of staff attitudes from the United States National Institute on Drug Abuse (NIDA) Clinical Trials Network indicated that personal beliefs regarding treatment affected clinical practice and the adoption of new interventions [20]. A survey of physicians' attitudes toward clinical guidelines or algorithms reported that physicians believed that guidelines were utilized more for cost containment purposes and less for quality improvement [21]. A more recent survey reported that substance abuse treatment professionals tend to have positive attitudes toward manualized treatment (*e.g*., beliefs that following a manual helps therapists evaluate and improve clinical skills, and enhances therapeutic outcomes) [22]. Further, increases in knowledge of the effectiveness of a medication and organizational support influence the use of a medication to treat substance dependence [15,23]. There is, however, a paucity of work about the specific organizational qualities that affect implementation of standardized assessments and practice guidelines.

Examination of utilization of practice guidelines and standardized tools may provide insight regarding agency and counselor factors associated with successful adoption for treatment planning and adaptations in service delivery. This study examines the organizational, staffing, and service characteristics that predict utilization of science-based practice tools and practice guidelines in outpatient substance abuse treatment programs.

## Methods

The New England Outpatient Survey [24] assessed the routine use of ASAM criteria for patient placement, use of treatment manuals (CSAT TIP documents; NIDA Therapy Manuals for Drug Addiction) and use of assessment tools such as the Addiction Severity Index [25] and the Beck Depression Inventory [26]. Organizational characteristics were assessed including agency size, funding sources, educational level, number of staff in recovery, and agency mission. Surveys completed in 1997, 1999, and 2001 assessed staff and agency characteristics, current practices, and innovations used in treatment. The 1997 and 2001 cohorts were examined in this paper.

### Participants

All licensed outpatient substance abuse treatment programs in the six New England states (CT, NH, ME, MA, RI, & VT) were included in the study sample. After accounting for programs that merged, moved or changed names, and programs operating multiple facilities, the list of potential outpatient centers included 341 organizations in 1997 [27]. Introductory letters were mailed, followed by a survey packet mailed one month later. Follow-up calls were placed to all treatment programs to confirm receipt of the packet, answer questions, draw attention to the study, and emphasize the importance of a timely response. The call concluded with scheduling an appointment with a research assistant for a telephone interview. Lost surveys were replaced and missed appointments were rescheduled as needed to ensure a representative sample. The full sample of respondents in 1997 (n = 281 agencies, 83% completion rate) consisted of directors or their representatives at freestanding substance abuse facilities (34%; n = 98), community mental health services (31%; n = 88), hospitals and integrated health care systems (18%; n = 51), and social service agencies and other types of programs (15%; n = 44).

The 2001 survey used similar sample selection including only outpatient programs that were licensed. Some agencies may have closed or merged, although study methods simply noted agencies that were no longer in the database without determination of the specific changes in status of facilities. The full sample of respondents in 2001 (n = 246 agencies, 72% completion rate) consisted of directors or their representatives at freestanding substance abuse facilities (26%; n = 64), community mental health services (20%; n = 50), hospitals and integrated health care systems (21%; n = 51), and social service agencies and other types of programs (33%; n = 81).

### Variables

Organizational characteristics and staffing were hypothesized to influence adoption of new technologies. All variables included in the structural models are transformations of items from the New England Outpatient Survey from 1997 and 2001 and are listed in Table 1. Models included three organizational variables (size of agency, managed care involvement, and being a freestanding alcohol and drug treatment program) and two staffing variables (staff recovery status and education level). Two service variables were included (counseling and ancillary services, and medical and medication services). The two outcome variables were use of practice guidelines and use of assessment and screening tools. The practice guidelines variable was a sum of the levels of care tools (*e.g*., use of ASAM Criteria, use of state modified placement criteria), treatment manuals, and protocols that are used in the organization. The screening or assessment tools measure was the sum of the standardized assessments used, including the Addiction Severity Index and the Beck Depression Inventory.

**Table 1:** Variable descriptions

Variable Name	Variable Description
**Organizational Items**	
Size of Agency	Number of unduplicated client visits over the period of one week in all outpatient units in the organization.
Managed Care Involvement	A 0-2 scale: one point was added if the agency received Medicaid managed care, and another point was added if the agency received private or commercial managed care.
Freestanding Clinic (non-hospital based)	A dichotomous variable that assigned a 1 if the primary mission was "substance abuse only facility" and a 0 for the rest of the clinics (hospital, primary care clinic or other clinic).
**Staffing Items**	
Counselor Education Level	Counselor education was measured with a weighted sum of the proportion of counselors with Doctoral, Masters, Bachelors, Associate or no degree: *i.e*., Educational Level = 4*(% of counselors with Ph.D.'s) + 3*(% of counselors with master's) + 2*(% of counselors with bachelor's) + 1*(% with less than a bachelor's). Higher scores indicate higher educational training for the staff.
Staff in Recovery	The percentage of staff at self-identified as in recovery.
**Services**	
Counseling and Ancillary Services	This item assessed the range of counseling services offered at the clinic. This included both counseling (*i.e*., psychotherapy, assessment, group therapy, psycho-educational groups, intensive outpatient, day and evening programs and case management support services) and support services (child care, employment counseling and transportation). This variable is scored as the sum of the services offered at each clinic.
Medical and Medication Services	This item assessed the range of medical (*i.e*., primary care, HIV testing, TB testing, OB/GYN, and pregnancy testing) and medication services (naltrexone, disulfiram, methadone, LAAM, TB medication, HIV medication, psychotropic, anti-depressants and anti-psychotics). This variable is the sum of each of the services offered at each clinic.
**Outcomes**	
Use of Assessment Tools	This was the sum of the standardized assessments for addiction that are used in the clinic (*e.g*., ASI, ADAD, DUSI, POSIT, LOCI, and RATE) as well as the psychiatric instruments used (*e.g*., SCID, GAF).
Use of Practice Guidelines	This was measured by items that assessed whether practice guidelines or other treatment protocols are used in the agency which included guidelines on medication management, depression, eating disorders, psychosis, alcohol, cocaine and heroin use disorders.

### Data Analysis

Structural equation modeling (SEM) with manifest (*i.e*., observed) variables was the primary analytic method. Standard regression models do not allow more than one criterion variable in the same model, and do not allow examination of mediating relationships between predictors. Structural equation modeling allows for complex interrelationships to be modeled simultaneously.

A maximum likelihood estimation method was used to estimate parameters using the CALIS module in the SAS Program [28-30]. Models were estimated for the 1997 and 2001 cohorts, respectively, with similar patterns expected for each time, but also allowing for differences over time. To identify the most parsimonious model, paths with non-significant regression weights or paths that did not contribute to a good model fit were dropped. Because SEM cannot handle missing data, only cases with complete data were available for analysis. A listwise deletion technique of cases with missing data resulted in 188 cases available for the 1997 model and 144 cases for the 2001 model. The variables in SEM included only manifest constructs. For example, "medical and medication services" is a construct of number of medical services and number of medication services (see Table 1). Because some manifest variables were constructed as sums of two or more indicators, and because some item wording changed between the two administrations, a standardization technique (z transformation) eliminated differences in the item metrics. Z tests of two proportions examined differences in binary variables and differences between means were assessed with ANOVAs. Some of these means were counts of variables. The F test is relatively robust and does not generate bias in response to violation of parametric assumptions provided that sample sizes are not overly discrepant.

## Results

Table 2 presents variable means and percentages from the full sample of respondents (1997 N = 281; 2001 N = 246). Actual sample sizes for each variable are presented in parentheses in each cell of Table 2. Significant increases were observed between 1997 and 2001 in the use of any practice guidelines, and in the mean number of medication and medical services offered (see Table 2). The ASAM Patient Placement Criteria was the most widely used level of care tool among participating outpatient treatment programs, with nearly half of the programs reporting the use of ASAM at both time points. Freestanding substance abuse treatment clinics were more likely to use ASAM (1997 = 63%; 2001 = 55%), followed by community mental health agencies (1997 = 55%; 2001 = 55%) and hospitals (1997 = 41%; 2001 = 52%). Use of ASAM placement criteria did not differ based on clinic size, licensure, or level of education.

**Table 2:** Comparisons of differences between the two time points of measurement

Variable^*a*^	1997 (N = 281)^*c*^		2001(N = 246)^*c*^			
	**Mean**	**SD**	**Mean**	**SD**	**Sig. tests**	**P-value**

% with Managed Care Involvement^*b*^	77.07% (266)	-	73.73% (217)		z = -.659	n.s.

% using Practice Guidelines^*b*^	66.65% (278)	-	75.33% (223)	-	z = 1.852	p < .10

% using Practice ASAM^*b*^	44.96% (278)	-	43.94% (217)	-	z = -.133	n.s

% of Staff in Recovery^*c*^	32.00% (266)	30.11	27.30% (221)	29.27	F(1,486) = 2.98	n.s.

Counseling Services (Number of)	4.93 (278)	1.52	5.17 (223)	1.38	F(1,499) = 3.18	n.s.

Medication Services (Number of)	0.49 (278)	0.99	2.82 (223)	1.94	**F(1,499) = 302.58**	**p < .0001**

Medical Services (Number of)	0.51 (278)	1.31	2.54 (223)	1.21	**F(1,499) = 509.03**	**p < .0001**

Ancillary Services (Number of)	0.54 (278)	0.94	0.69 (223)	0.89	F(1,499) = 3.48	n.s.

Clients served per week	119.00 (272)	238.03	189.80 (221)	925.36	F(1,490) = 1.47	n.s.

^*a *^Value in parentheses is the n for each variable.

^*b *^This designates a binomial variable. The percentage is the number of clinics that have this characteristic. Test statistic used was a z test of two proportions.

^*c *^This is a mean of the percent of staff in recovery for each clinic.

The most common standardized assessment tool in the participating outpatient treatment programs was the Addiction Severity Index (1997 = 33%; 2001 = 29%). The most common mental health assessment was the Global Assessment of Functioning (1997 = 79%; 2001 = 84%), followed by the Structured Clinical Interview for Diagnosis (SCID), (1997 = 32%; 2001 = 40%). Many programs used assessments created specifically by their staff to assess addiction and mental health (1997 = 100%; 2001 = 86%).

**Figure 1:**
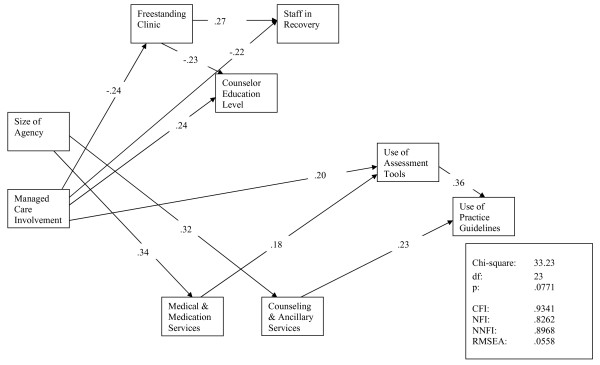
**Time 1 Path Model of Predictors of Practice Guidelines and Practice Tools**.

The structural equation model for the 1997 cohort is presented in Figure 1 and is based on 188 complete observations. This model demonstrated good fit indices (*X*^2 ^= 33.23, df = 23, p = .0771; CFI = .9341, RMSEA = .0558). No residuals were greater than 2.22. Although not temporal, the models represent relationships among variables. In this first model, programs that have greater involvement with managed care and programs that offer more medical and medication services are more likely to use assessment and screening tools. Extending these relationships, the model also captures the relationships between the increased use of screening and assessment tools, and provision of counseling and ancillary services, and the greater use of practice guidelines.

Programs with more managed care involvement were less likely to identify themselves as a freestanding addiction treatment programs (as compared to multi-service, hospital, or medical based clinics). Programs with more involvement from managed care were also more likely to have staff with advanced degrees, as opposed to freestanding programs that employed more staff in recovery. Larger agencies were more likely to provide a range of treatment options including offering medications, medical services and additional ancillary or support services.

**Figure 2:**
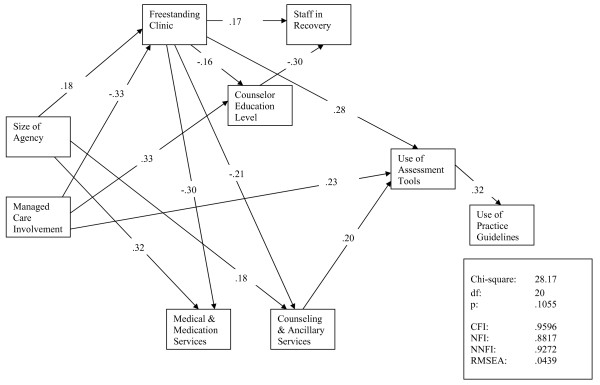
**Time 2 Path Model of Predictors of Practice Guidelines and Practice Tools**.

A similar model with organizational, staff, and service delivery variables was constructed for the 2001 cohort and is based on 144 complete observations (see Figure 2). Eliminating non-significant paths and high residuals reduced this model to a parsimonious description of the interrelationships of these variables. The data fit the model well (*X*^2 ^= 28.17, df = 20, p = .1055; CFI = .9596, RMSEA = .0439) and no residuals were greater than 2.50. Managed care involvement also was influential in this second model. Freestanding clinics or treatment programs were less likely to be supported by managed care funding. Managed care also was associated with higher counselor education levels and use of assessment and screening tools. Similar to the 1997 model, agencies that were larger in size again offered more medical services, medications and a greater range of counselling and ancillary services.

## Discussion

The results of this study suggest that the two outcomes, use of standardized assessment tools and use of practice guidelines, are interdependent. In the first model (Time 1), managed care involvement, and provision of medical care services and use of medications had the strongest association with increased use of standardized assessment tools. Use of assessment tools and offering counseling and ancillary services was then associated with greater use of practice guidelines. In the second model (Time 2), again managed care involvement was associated with increased use of assessment tools, as was being a stand-alone drug treatment agency. The relationship between provision of counseling and ancillary services had a stronger association with assessment tools in this model, but the relationship between the use of assessment tools and a greater use of practice guidelines remained strong in the second model. Thus, programs that offer a larger range of services (*i.e*., medical, ancillary or wrap around, medication and counseling) and have strong relationships with or receive funding from managed care entities, appear to be more likely to adopt standardized assessments and practice guidelines or algorithms.

In terms of clinical staff and their impact on adoption, programs that were free-standing or non-hospital based were also more likely to have a greater number of staff in recovery and providers with fewer years of education. Managed care involvement was also associated with having more staff with higher education.

It is also interesting to note that the majority of agencies created their own assessments/intakes, or possibly used a mix of standardized assessments, suggesting a legacy of program tools that may not be updated frequently. This range of eclectic assessment practices may also reflect dissatisfaction with, or a lack of knowledge about validated assessment tools. Thus the models from this study provide data for state, county and community policy makers to consider when allocating resources, planning trainings, and implementing targeted systemic interventions.

### Adoption of Evidence-Based Practices

Overall, research thus far suggests limited use of practice guidelines in community-based substance abuse treatment. To better understand why this implementation or such clinical decision guides is slow, organizational, environmental and staff factors must be examined. To date, services research suggests that the decision to adopt new practices is often influenced by the demands of the funding entities and institutional structures [14,15]. Further, providers that are most likely to adopt new practices tend to have more formal education, come from a higher social status, and have a more favorable outlook regarding change [17]. These early adopters are also more likely to take the initiative to seek out information and have a greater number of national professional contacts [17]. Clinicians indicate that they are more open to new strategies when those practices clearly improve services and outcomes, are consistent with their philosophy of treatment, and are supported with training that includes observation, practice, supervision, and feedback prior to full adoption [31-33]. Organizational structures and practices (*e.g*., funding mechanisms, agency mission, and data management systems) also impact the use of innovations within substance abuse treatment programs. Thus, both individual treatment providers and agency level or organizational characteristics influence the implementation of new practices including practice guidelines and the use of assessment and screening tools [15,18,19]. The results of this study correspond with this implementation science and classical diffusion theory literature. Funding mechanisms (involvement with managed care), treatment philosophy (counseling and use of ancillary services), and staff characteristics were associated with greater implementation of practice guidelines and assessment tools. The ASAM patient placement guidelines and the ASI assessment tool were widely used and were considered best practices by the participating outpatient treatment programs.

Interestingly, although having greater involvement with managed care was associated with specific staff characteristics as well as with use of assessment tools and practice guidelines, in this project the level of counselor education in the programs and the number of staff in recovery was not directly associated with greater use of practice guidelines. Although other research has found that education is associated with more positive opinions about evidence-based practices [32] and greater acceptance of innovation [17], previous investigations were based on attitudes surveys with treatment center staff. The current data came from a survey of treatment center directors and not a direct assessment of counselors or staff. Methodological differences may explain the difference in findings. It may also be that use of assessment tools and practices guidelines present a greater level of controversy and challenge for providers. According to Miller, Zweben and Johnson [34], retraining providers with established habits and experiences is both challenging and time consuming. The use of standardized assessment tools and practice guidelines requires considerable training and supervision. Autonomy also has been associated with increased satisfaction and commitment to the organization [35], thus providers may value independence in decision-making and they may also not have access to the necessary training and supervision related to assessment and the use of guidelines. Finally, staff may have felt constrained by guidelines and may not have perceived any clinical or organizational benefit with their implementation in their treatment programs. Thus, the findings from this study may differ somewhat from previous studies that suggest counselor characteristics predict adoption of new practices due to methods (*i.e*., response of agency directors), the amount of training and supervision required to use standardized tools and guidelines, as well the constraint or lack of autonomy that counselors may resist when using use tools/guidelines.

Findings from the current study also suggest an important relationship between agency size, counseling and ancillary services, and use of practice guidelines and standardized assessment tools. Larger agencies offered more counseling and ancillary services which contributed to the use of practice guidelines and assessment/screening tools. Integration of primary care and mental health with substance abuse treatment offers increased access to a range of providers, medications, and independence from government funding which provides leverage for change and flexible, progressive decision-making for the organization [36,37]. Large agencies that offer a wider range of services have the resources to purchase assessment instruments, provide training, conduct organizational change processes, and flex their resources in a fashion that is necessary for the implementation of standardized practice guidelines. Consistent with the literature, participation in ancillary service delivery appears to connect programs with other community providers, exposing managers and counselors to diagnostic tools and procedures outside their immediate organization [38]. Applying this literature base to specific clinical practices, including use of standardized assessment tools and practice guidelines, provides greater understanding of these factors and their dynamic and interdependent impact on diffusion of innovations

### Limitations

This study had a number of limitations. The sample included six New England states, which may have limited generalizability to the rest of the United States. New England is a region with high managed care penetration and a high degree of public sector treatment agencies which may not be characteristic of the rest of the country. In addition, slight wording changes in the surveys between the two data collection points created some restrictions regarding the type of longitudinal analysis that could be used (*i.e*., models are not dependent time 1 to time 2). Had the models been able to be dependent, statistical tests would likely have been more sensitive. However, because not all clinics were represented in each sample, this was not possible and is a limitation of the study. Missing data imputation is not used with structural equation modeling, thus a listwise deletion technique results in parsimonious models based on cases with complete data available for analysis. Finally, although data were collected in 2001, dissemination of these findings is important because they are consistent with the literature confirming the difficulties changing practice patterns and disseminating research findings. The study results may also be used for comparison with future assessment that evaluates the use of practice guidelines.

## Conclusions

This study provides managers, clinicians and policy-makers with a framework for understanding factors related to the adoption of new technologies in substance abuse treatment. Findings from this study suggest that increasing the use of evidence-based practices or tools may require provider and organizational change efforts as well as systems wide interventions that result in revised policies, changes in contract language and specific detailed descriptions of procedures and expectations. State substance abuse authorities (SSAs), regulatory and funding entities clearly play a significant role in clinical practice and the quality of addiction treatment services. Expanding the framework from provider and organizational factors to include state/county and city policy makers, current change efforts such as the Robert Wood Johnson *Advancing Recovery Initiative *are emerging. This intervention partners the state authority with providers and other stakeholders to address systems wide change aimed at increasing the use of evidence-based practices. Through this type of collaboration, and with appropriate state administrative data, research literature, and leadership, policy makers will be able to make informed decisions that impact the quality of addiction services.

## Competing interests

The authors declare that they have no competing interests.

## Authors' contributions

TR participated in conceptualization of this paper, the statistical analysis interpretation, and helped to draft the manuscript. BEF participated in the statistical analysis and interpretation. GAS helped to draft the manuscript. DM conceived of the study and participated in its design and implementation. All authors read and approved the final manuscript.
